# The Draft Whole-Genome Sequence of the Antibiotic Producer Empedobacter haloabium ATCC 31962 Provides Indications for Its Taxonomic Reclassification

**DOI:** 10.1128/MRA.01120-19

**Published:** 2019-11-07

**Authors:** Henrike Miess, Patricia Arlt, Alexander Kristian Apel, Tilmann Weber, Kay Nieselt, Friederike Hanssen, Stefan Czemmel, Sven Nahnsen, Harald Gross

**Affiliations:** a Department of Pharmaceutical Biology, Pharmaceutical Institute, University of Tübingen, Tübingen, Germany; b The Novo Nordisk Foundation Center for Biosustainability, Technical University of Denmark, Kongens Lyngby, Denmark; c Integrative Transcriptomics, Institute for Bioinformatics and Medical Informatics, University of Tübingen, Tübingen, Germany; d Quantitative Biology Center (QBiC), University of Tübingen, Tübingen, Germany; e German Centre for Infection Research (DZIF), Partner Site Tübingen, Tübingen, Germany; University of Maryland School of Medicine

## Abstract

Strain ATCC 31962 was formerly taxonomically classified as Empedobacter haloabium and reported to be the producer of the lipopeptide antibiotic empedopeptin. Here, we report the draft genome sequence of ATCC 31962, which encodes regions that suggest a distinct biosynthetic capacity and suggests its taxonomic reclassification.

## ANNOUNCEMENT

Empedobacter haloabium ATCC 31962 (syn. designation G393-B445) was isolated in the early 1980s from a soil sample collected in Yamate-Dori, Tokyo, Japan, and was suggested to be a new species of the genus *Empedobacter* (1). It exhibited antibacterial activity in a screening panel and was found to produce the highly potent antibiotic empedopeptin (syn. BMY-28117 and Bu-2517) (2, 3), which acts as a cell wall biosynthesis inhibitor by complex formation with peptidoglycan precursors (4). In order to investigate the complete biosynthetic capacity for secondary metabolism, to locate the biosynthetic gene cluster of empedopeptin, and to clarify the taxonomic position of the strain for safety reasons, the genome of ATCC 31962 was sequenced.

Strain ATCC 31962 was grown in 20 ml meat medium (2% soluble starch, 1% glucose, 0.2% meat extract, 0.2% Bacto yeast extract, 0.5% N-Z-Case, 0.2% CaCO_3_, and double-distilled water [ddH_2_O] [pH 7.0]) for 2 to 3 days at 27°C on a rotary shaker (140 rpm). For genomic DNA isolation, the Qiagen genomic DNA purification kit was used in combination with 100/G Genomic-tips according to the manufacturer’s protocol, except that for the bacterial lysis, the handled volumes were doubled, and the incubation time at 50°C was prolonged until a clear lysate was obtained.

The whole-genome sequence of ATCC 31962 was obtained using a combined strategy of paired-end sequencing (NEBNext sample preparation kit, 2 × 76 bp) with an Illumina GA IIx instrument and mate pair sequencing (Nextera mate pair sample preparation kit v2, 151 bp) with an Illumina MiSeq instrument. Subsequently, FastQC v0.11.2 (5) was used to check both libraries for adapter content and base quality. From the paired-end library, 47,704,862 reads of 75 bp were obtained. FastQC identified adapter contamination, and therefore reads were adapter clipped, and the remaining 41,254,498 reads were subject to *de novo* assembly. Within the frame of the mate pair approach, 3,395,400 paired-end reads of 150 bp were sequenced. FastQC did not reveal any adapter content, and base quality was high, so all original reads were subjected to the second assembly. In both cases, the reads were assembled using SOAPdenovo v.1.05 (6, 7). The contigs of both libraries were combined and then subjected to the string-based assembly of MADAM v.beta-version:06-2013 (8). Default parameters were used for all software mentioned above, except that k = 63 was used for the library with a small insert size and k = 109 for the library with a large insert size. Overall, the final assembly yielded a 6,678,028-nucleotide draft genome at 102-fold coverage, consisting of 106 contigs (*N*_50_, 132,565 bp; *L*_50_, 18) in total. The *in silico*-determined G+C content of 66.5% is in full agreement with the experimentally determined value of 66.5 ± 1.5% (1). The assembled contigs were annotated with the NCBI Prokaryotic Genome Annotation Pipeline (PGAP) v4.8 (9), yielding a total of 5,690 predicted protein-coding sequences.

A BLAST analysis of the partial 16S rRNA of strain ATCC 31962 (GenBank accession number NR_125708) identified *Massilia* sp. strain YMA4 (KX444135) and Massilia armeniaca ZMN-3 (CP028324) as the closest cultured representatives, both sharing 99.5% 16S rRNA gene sequence identity with ATCC 31962. Using autoMLST (10), the overall genome analysis was shown not to support the initial taxonomic classification of the strain as an *Empedobacter* species. According to an average nucleotide identity (ANI) analysis, the closest related strains are *Massilia* sp. strain NR4-1 (ANI, 83.6%) and Rugamonas rubra ATCC 43154^T^ (ANI, 83.5%).

### Data availability.

This whole-genome sequencing (WGS) project has been deposited at DDBJ/ENA/GenBank under the accession number VPFC00000000. The sequencing reads have been deposited under the accession numbers SRX6060211 and SRX6060212. All reads have been deposited in the SRA and are associated with BioProject number PRJNA532449.
